# The Cranial Characteristics and Powers of Human Races

**Published:** 1854-08

**Authors:** S. B. Hunt

**Affiliations:** Buffalo


BUFFALO, June 16th, 1854.

DR. S. B. HUNT:

Sir: In pursuance of a resolution to that effect, adopted at the semi-annual Meeting of the Medical Society of the County of Erie held the 13th inst., I solicit a copy of your Oration, delivered on that occasion, for publication in the Buffalo Medical Journal.

Yours Respectfully,

JAMES M. NEWMAN,

Secretary Med. Soc. Erie Co.

ART. III.—*The Cranial Characteristics and Powers of Human Races.* An Oration read before the Medical Society of the County of Erie at its Semi-Annual Meeting, held June 13th, 1854.

At a period like the present, when the rapid overspreading of the North American continent has suddenly given rise to a new code of national policy; when the necessities of new events have added the phrase "manifest destiny" to the vocabulary of the statesman; when all politicians have become ethnologists, and talk learnedly of the antipathies and affiliations of races: when an American Secretary of State brings up the diversity of races in a diplomatic discussion, as a sufficient reason for a peculiar line of national action; when we behold prophecies made to-day, and fulfilled to-morrow, of some new conquest for the Anglo Saxon; when a process of emigration, which is rather an Exodus of a whole people, than a colonizing of a part; together with an increased prolificacy, owing to a lengthened average of human life, is spreading a new and exotic race over our continent, displacing from their territories the old inhabitants—when all these concurrent circumstances are thus operating, it is fitting that the physician and the anatomist, should bring forth from their store of acquirement those great anatomical conditions which are the cause of all this turmoil.

It is not necessary for us to discuss the ultimate unity or diversity of the human race. Whatever may have been man's primeval condition, he is *now* divided into races, no less by anatomical differences, than by degrees of intelligence, and civilization. And it is my sincere belief that these differences are permanent; that whatever may be the *actual* improvement of any, or all races, their *relative* position must remain the same. Said Dr. Robert Knox: "Human history cannot be a mere chapter of accidents. The fate, of nations cannot be always regulated by chance; its literature, science, art wealth, religion, language, laws and morals, cannot surely be the result of mere accidental circumstances."

It should not be objected to this that it inculcates the doctrine of inequality of races. The notion of actual equality is Utopian; it does not exist in fact—it is both unscientific, and unscriptural. In looking back upon the workings of that providence which writes the pages of human history we find one nation always in servitude, another always free—one particular family gradually overspreads the temperate zone; before it perishes all other kindred, tribes, and tongues.

Upon the North American continent we have the representatives of the great Caucasian family, of the aborigines of the soil, the negro, and, more recently, a Chinese emigration has sent the Mongol to our Pacific shores.

Let us study a little the relative anatomy of these races. The most marked and essential difference which separates one human type from another, is in the capacity, and shape of the cranium. All other variations seem secondary to this. Among the subdivisions of the great families now dwelling on this continent, we have, first, the Teutonic races, with an average cranial capacity of 92 cubic inches; second, the Celtic, with 87 inches; the Chinese, and the Negro, each, 83; the Barbarous tribes of American Indians 84; and the Toltecan family 77 cubic inches. Among the remarkable varieties which this list presents, we find that the German average is 90, the

Anglo-American 90, and the English 96 cubic inches. The low measurement of the American, as compared with his English progenitors, is owing to the fact that only skulls of men remarkable for crime were included in it by Dr. Morton; from whose tables we obtain these results; while, in all other instances, we have merely a casual selection of all varieties of character. Another fact worthy of notice, is, that the measurement of the negro is greater than that of the Toltecan, and equal to the Mongolian average. Another feature in the negro skull is the fact, that American born negroes have 1 cubic inch less than the native African families.

It will be seen from this brief statement, that the mere capacity of the skull is not, alone, the true index of ability; while, at the same time, it leaves untouched the great fact, that the Teutonic, the family of largest cranial development, is the most advanced in civilization, and the most vigorous in growth, and conquest. The secret of the relative force of nations, where cranial capacity does not fully account for it, must be found in the other condition alluded to, the relative size of the intellectual lobe. Certain varieties exist in the human skull which modify this relation. Thus the Teuton has a forehead, high, broad, full, and nearly vertical, the coronal region is well developed, and the occiput is well rounded, but has no excess over the anterior region. Departures from this ideal, will, as they gradually assume the prognathous form, determine the relative intellectuality of races.

Let us trace now the progress of those races which are known to the history of the North American continent.

The monumental history, as well as the traditions of the aborigines of our country, indicate that the Toltecan, or Peruvian, was once the dominant race of this continent. As described by Cortez and his followers, they were a gentle people, of fixed habits, given to assembling in large communities, and the building of great cities. The arts of civilization existed among them to a great extent. A monarchical government, a priestly hierarchy, and a provident agriculture indicated a condition far above barbarism. Their average cranial capacity, as ascertained by Morton from the measurement of 213 skulls, was 77 cubic inches. Its conformation presented a low receding forehead, the longitudinal and parietal diameter nearly equal, a flattened occiput, high cheek bones, and heavy and projecting jaws. This race once held possession from the great lakes to the isthmus of Darien. It was they who constructed the forts and mounds which dot our western praries. But long before the peopling of North America by the whites, they had disappeared from the whole country north of the Rio Grande; and their place was occupied by a race superior to them in cranial development, but inferior in the arts. The Barbarous tribes had some 7 cubic inches of brain the advantage over the Toltecans. The cranial conformation was similar, with the exception of a fuller occiput, and smaller intellectual lobe. These anatomical characters formed an analogue in their minds. Crafty, subtle, vindictive, nomadic, despising manual labor, and incapable of civilization, they were still permitted, in the Providence of God, to drive before them the mild Toltecan, and give to rapine and blood the land which once waved with corn. It was the manifest destiny of the Toltecan race to perish from the earth. Their civilization, their knowledge of fortification and defence, were no match for the larger brain of the red man. The men of largest brain, of strongest will, fiercest animal passions, and smallest share of human sympathies, passed from their north-eastern origin, and swept all obstacles from their path. It was a work of annihilation, and nothing was left of the Toltecan but his forts and mounds.

The second act in this great drama opens the most important and immense migration of the human race on record. There came to the shores of New-England and Virginia some feeble bands of men, who, whether rightfully or not, were soon engaged in bloody wars with the numerous tribes around them. Looking at the probabilities as they then existed, the chances were a thousand to one that, a broil once commenced between the white and the red man, the former would soon be driven from the shores of the continent, or find a grave beneath its forests. They had to contend with a race numerous, powerful, vindictive, armed with efficient weapons, and the bravery to use them. Why is it, then, that we have seen the Teuton gradually enlarging his borders, and the red man as steadily perishing before him? The work is like that which the Indian had previously inflicted on the Toltecan. It was not conquest or subjection, but annihilation. Rank by rank, and tribe by tribe, the red man faded from his possession. Like some Sarsar wind of death the races of the Teuton have passed from the portals of the East, until now the golden shores of the Pacific acknowledge their dominion. It mattered little what means were chosen to accomplish this result. The peaceful policy of William Penn, and the stern unyielding integrity of the Puritans, were as fatal to the Indian as the fierce slaughter of the Spaniards in the halls of Montezuma. And the high necessities of civilization were but a secondary element in this contest. On the whole line of advance, from the Bay of Massachusetts to the Gulf of Mexico, the progress of the white race was preceded, and pioneered, by a class of adventurers who fled from the life of towns, and assimilated themselves to barbarism. It was not for civilization that the Daniel Boones of our country fought and struggled.

They contended with the Indian for his hunting grounds, and not for sites of cities. It was the physiological antipathy of race for race, not sufficiently proximate, and too proud and stubborn to blend.

And here we may pause to notice another marked difference in the conquering races. The Teuton, with an average cranial capacity of 92 inches—or if we take the pure English standard of the Puritans, of 96 inches, making a capacity of 12 cubic inches above that of the red man, fought less, and conquered more than did the Spaniards and French at the south, with an average of 84, and 87 cubic inches; thus nearly assimilating them to the Barbarous, but not reducing them to the Toltecan, measurement. As a natural consequence we find that the Teuton has never widely amalgamated with the Indian. The animal passions were too feeble, and the innate pride of birth and connection too high, for such an intermingling. But the converse held true with the Spaniard and Frenchman. The Iberian and Celt belong to the swarthy families of the Carcasian race, and are as distinctly seperable from the Anglo-Saxon, as from the negro. Possessing as a race five cubic inches less of brain than the Teuton, they more nearly approximate the aborigines than the men of the north. They have every where first fought and conquered, and then amalgamated with the Indian. The consequence is a feeble and hybrid race; defining hybridity as a loss of permanence of national type. The physical degeneration which has resulted from this blending, is a very noteworthy feature in anatomical science. The races now inhabiting Mexico are a breed, so disgracefully mixed and intermingled, that the types of the heroic Indian, as well as the dignified Spaniard, have alike disappeared. The average size of the head in Mexico is so small, that it is with the greatest difficulty, that an American, of average cranial size, can find a native hat sufficiently large.

Still another race comes in to mingle in the confusion of American population. We are indebted to our English forefathers, for the presence among us of more than three millions of a low type of human organization—the Negro. Prognathous jaws, narrow elongated forms, receding foreheads, large posterior development, and an internal capacity of only 83 inches, characterize the cranium of the African negro. The cranial capacity is nine inches less than that of the Teuton, but still exceeding the Toltecan by 6 inches, and only 1 less than the Barbarous Indian. There is, therefore, nothing in the mere size of the negro skull which especially marks him for servitude, or renders impossible a certain degree of civilization. Although he has never, in his native state, attained to any degree of culture, he is endowed with a wonderful imitative faculty, which enables him to adapt himself to the customs of civilized life. But we find that he more readily amalgamates with the Indian, than with the white The red man, though he sometimes makes a slave of his black fellows, is still more generally disposed to admit him to a footing of equality. In his relations with the whites, he has now for two centuries remained in servitude, without an effort, on his part, to escape from bondage. The casual flight of a few solitary individuals does not invalidate the fact that he is enchained by a people which could not thus enslave the Indian. The story of Uncle Tom's Cabin contains a most truthful moral on this point, however unconsciously on the part of the author. "George," the almost white slave, strikes for freedom with a bold hand; preferring death to slavery. So, too, did "Cassy," and every other light mulatto in the book. But we find that Mrs. Stowe has always portrayed the pure black as a willing bondsman, and "Uncle Tom," himself, as a model of submission to the lash, and to bitterest wrong and outrage. This was not mere Christian non-resistance. The meekest martyr, from St. Stephen to John Rogers, would have resisted such wrong, by force of arms. It is an inborn characteristic of the black race.

While I would not sanction the idea that the mere fact of inferiority, or diversity, of race, can justify the holding of a fellow man (for a fellow man he is) in involuntary servitude, I still think, that the anatomical facts of difference should have some influence in modifying our sentiments, and render us slow in imposing the responsibility of self-support upon a raçe, whose ability to maintain themselves, in competition with the white man, is at least as much a problem, as is that of the co-existence of the Anglo-Saxon, and the Indian. It is impossible for 83 cubic inches of cerebral matter, fed by negro blood, to compete with 92 of educated, Tentonic, brain. It is not the province of the anatomist to decide what should be done; but it is safe to assume, that any being, however degraded, if he possesses reason and conscience, should also possess the liberty to use them for his own welfare. The limit of authority over a degraded race should not extend beyond an exercise of paternal care and superior wisdom, in guiding, protecting, and elevating it, in such a manner of life as is best fitted to its capacities.

The amalgamation of the two races produces the mulatto, who manifests a certain degree of hybridity. He is a superior negro, but a very inferior white man. As we go on approximating to the white, we have increasing aptitude to learn, and greater intelligence; but this is accompanied by a corresponding degradation of the white. The mulatto is an unnatural and a sinful existence. Feeble in constitution, unable to perform severe labor, he manifests a tendency to scrofulous disease, and early death. Though the pure negro is naturally long lived, we find the mulatto rarely attaining the verge of old age. It is a notorious fact, that, were it not for constant importations from the south, the race of negroes would soon disappear from the northern states, from amalgamation, and consequent short life. If amalgamation is thus fatal to the existence of the negro, what better would be his condition if left to his own resources. It is but just that we should look the anatomical argument fairly in the face. The condition of the negro has ever been that of servitude—a consequence of his lack of brains. It cannot be pretended that this should form a justification of American Slavery; but the anatomist will still shrink from hastily disturbing the present order of things. An immediate setting free of the bondsmen of the south, would place three and a half millions of an inferior race in competition with one far superior to it in anatomical perfection. Who can doubt where misery would fall? The experiment has already been twice tried on this continent. The Toltecan and the Indian have in turn faded, and passed away from the broad lands they once claimed as their own. Without a claim to the soil, without a vestige of national organization, and in competition with a vastly superior race, that annihilation which has so surely dogged the retreating footsteps of the Indian, would find but a feeble resistance from the humble crouching African.

One circumstance may, in this contingency, operate in favor of the negro. Had the Indian been capable of subjection to slavery he would still be found among us. The negro would soon, in freedom, adapt himself somewhat to his new condition; and, although a large class might, like the wretched inhabitants of the British West Indies, prefer abject poverty to labor, yet the influence of a colder climate, and the necessity of providing for a winter, might gradually engraft industrious habits. Even the ever working bee, when transported to Jamaica, laid up his store of honey for a single season only. Ever after that, he forgot his provident northern notions, and led a roisterous and dissipated life among the sweets of the sugar houses, unmindful of the morrow.

It is now a received opinion with ethnologists, that the large headed Teuton is the dominant race of all the earth. Wherever climate will permit his existence, his passion for discovery leads him. The negro, the Hindostanee, the Malay, the aborigines of America, have all fallen before him; and now he knocks at the door of the Japanese Mongol, and demands admission there. One by one the lesser tribes have owned his sway. The lively Celt of Ireland has yielded his long fought battle with the English Teuton; the high spirited Hungarian, and the wily Italian, feel the yoke of the Austrian Teuton; and still another family of this great group is pressing down upon the Turk. The stream of emigration has filled the United States with unceasing additions of Teutonic blood. Before them has fled the Indian, and beneath their iron rule is bowed the unfortunate African.

Who can tell where, or when, this conquering advance shall cease? What shall be the fate of feebler nations beneath its sway?

In these great facts, this hastily gathered evidence of "manifest destiny," we read a lesson which politicians well might learn. The doctrine that "all men are created free and equal," is an anatomical, physiological, and Scriptural impossibility. So long as one star differeth from another star in glory, so long as the infinite gradations from dust to Deity rank one above another, so long as man is but a little *lower* than the angels, while God is over all, so long shall the difference which He has implanted in the human race, remain, unchanged in type, though progressive in excellence, until the last act of the drama shall open upon a single dominant race, in a world whence all others shall have disappeared.

